# Genomic analysis of the main epidemiological lineages of *Acinetobacter baumannii* in Mexico

**DOI:** 10.3389/fcimb.2024.1499839

**Published:** 2025-01-10

**Authors:** Barrios-Camacho Humberto, Lozano-Aguirre Luis, Duran-Bedolla Josefina

**Affiliations:** ^1^ Centro de Investigación Sobre Enfermedades Infecciosas (CISEI), Departamento de Diagnóstico Epidemiológico, Instituto Nacional de Salud Pública (INSP), Cuernavaca, Mexico; ^2^ Programa de Genómica Evolutiva, Centro de Ciencias Genómicas, Universidad Nacional Autónoma de México, Cuernavaca, Mexico

**Keywords:** *A. baumannii*, genomic analyses, beta-lactamase, international clones, sequences type

## Abstract

*Acinetobacter baumannii* has emerged as a critical global health threat due to its exceptional survival skills in adverse environment and its ability to acquire antibiotic resistance, presenting significant challenges for infection treatment and control. The World Health Organization has classified carbapenem-resistant *A. baumannii* as a “Critical Priority” pathogen to guide research and the development of control and prevention strategies. Epidemiological surveillance methodologies provide the tools necessary for classifying *A. baumannii* into international clonal lineages, facilitating the analysis of molecular characteristics, global dissemination, and evolution. This study provides a detailed analysis of the molecular epidemiology of *A. baumannii* in Mexico, focusing on identifying the main international clonal lineages. Genomic analyses of 146 genomes, along with information from previous studies, identified 24 different sequence types according to the Oxford Scheme. The major international clone IC2 (CC208) was identified and harbors β-lactamases OXA-66, ADC-30, OXA-72, and is predicted to possess the OCL1 locus. The international clone IC5 (CC205) carries β-lactamase OXA-65, along with ADC-214 and OXA-239, with OCL10 predicted in 82.2% of the genomes. The international clone IC7 (CC229) harbors β-lactamase OXA-64, as well as ADC-174 and ADC-214, with OCL6 and OCL7 loci predicted. These international clones were identified in different periods and regions of Mexico and are likely to be widely distributed throughout the country. The analysis of each lineage reveals distinct molecular characteristics, including sequence types, capsule typing, outer core loci, and specific antibiotic resistance profiles. Understanding these features is crucial for elucidating their roles in infection dynamics, resistance mechanisms, and their impact on clinical outcomes.

## Introduction

*Acinetobacter baumannii* is a non-motile, aerobic Gram-negative bacterium that has become a major cause of healthcare-associated infections (HAIs) worldwide. In 1971, *A. baumannii* was regarded as a pathogen of minimal clinical significance and was susceptible to all antibiotics (Fournier et al., 2006). However, the overuse and misuse of antibiotics have accelerated the development of resistance, leading to the emergence of multidrug-resistant (MDR) and extensively drug-resistant (XDR) clones that are difficult to treat and eradicate. Today, *A. baumannii* is intrinsically resistant to penicillin and has acquired resistance to a wide range of antibiotics, including fluoroquinolones, cephalosporins, and carbapenems, which are considered last-line treatments for severe infections. Since 2017, the World Health Organization has designated carbapenem-resistant *A. baumannii* as a “Critical Priority” pathogen, highlighting the urgent need for focused research and novel strategies for prevention, control and treatment (WHO, 2017, 2024). *A. baumannii* could persist for extended periods in the environment, on inert surfaces, and on medical equipment contributes to the persistence and spread of multidrug-resistant clones. The primary mechanism of carbapenems resistance in *A. baumannii* is the presence of oxacillinases (OXA) genes. The *bla*-OXA-51-like group includes several closely related variants, such as OXA-64 and OXA-65, which are particularly useful for typing due to their high discriminatory ability, especially when combined with other techniques (Shelenkov et al., 2023). Molecular epidemiology tools such as Multi-Locus Sequence Typing (MLST) are essential for characterizing genetic differences among bacterial isolates. By classifying isolates of *A. baumannii* according to their sequence type (ST), MLST creates clonal complexes (CC) from isolates with comparable genetic backgrounds. This method helps identify predominant isolates in different geographical regions, understand clonal expansion, and reconstruct evolutionary relationships (Shelenkov et al., 2023). In Mexico, *A. baumannii* has become a significant concern due to its high resistance to multiple antibiotics and its association with hospital outbreaks (Bocanegra-Ibarias et al., 2015; Gonzalez-Villoria et al., 2016; Tamayo-Legorreta et al., 2014, 2016). Genomic analysis has revealed the presence of various STs related to International Clones (IC), indicating possible global dissemination of these resistant isolates. Notably, ST-205 and ST-208 are among the primary sequence types identified, correlating with high-risk international clones (Alcántar-Curiel et al., 2019). This highlights the urgent need for continuous surveillance and targeted interventions to mitigate the spread of these resistant pathogens. Whole genome sequencing (WGS) has provided a comprehensive view of genomic diversity and evolutionary dynamics using the antibiotic resistance profiles, pathogenicity genes, including lipooligosaccharide outer core loci (OCLs), which complement MLST data (Wyres et al., 2020). In this study we present a comprehensive analysis of the molecular epidemiology of *A. baumannii* reported in Mexico. Our findings reveal the significant prevalence of specific clonal complexes (CCs) related to four major international clones (ICs) across different regions. The results will provide critical insights into the clonal lineages of *A. baumannii* in Mexico and their global context, enhancing public health strategies and strengthening surveillance systems in healthcare settings.

## Materials and methods

### Identification and selection of *A. baumannii* genomes

We analyzed 146 A*. baumannii* genomes from Mexico, identified up to July 2024, obtained from the NCBI GeneBank and RefSeq databases using the ncbi_datasets program v14.24.0. Genomes that were incomplete or lacked proper annotation were excluded from the analysis. These genomes were from eight of Mexico’s 32 states, spanning isolation years from 2006 to 2021. Although many of these genomes have been previously reported, we included all available genomes to provide a comprehensive overview on the molecular epidemiology of *A. baumannii* in Mexico. This approach allows us to identify the most prevalent sequence types (STs) and international clones (ICs) within the country. In addition to the genomes, we also incorporated data from 51 previously reported isolates, resulting in a total dataset of 197 isolates from 35 different hospitals (**Supplementary Dataset 1**).

### Average nucleotide identity and heatmap

The average nucleotide identity (ANI) analysis for these 146 genomes was performed using PYANI software v0.2.12 with the ANIm alignment method (Pritchard et al., 2016). The resulting ANI heatmap was generated with the R pheatmap package (version 1.0.12) in RStudio, utilizing the ANIm_percentage_identity.tab file.

### Core genome and phylogenetic analysis

We identified the core genome of the 146 A*. baumannii* genomes using GET_HOMOLOGUES software (version 11042019) with three methods: bidirectional best-hit, COGtriangles, and OrthoMCL clustering algorithms (Contreras-Moreira and Vinuesa, 2013). This yielded 1,590 clusters of orthologous groups, each containing one gene per genome. Phylogenetic analysis was inferred using Get_Phylomarkers software (version 2.2.8) (Vinuesa et al., 2018), using 259 marker genes from the core genome to create a concatenated alignment. The *A. baumannii* phylogenetic tree was constructed using RAxML (version 8.2.10) with the GTR evolutionary model (Stamatakis, 2014) and visualized using MEGA software (Tamura et al., 2011).

### *In silico* predictions of the AR genes, OCL and KL locus

Antibiotic resistance genes were analyzed with the Resistance Gene Identifier (RGI) integrated into the Comprehensive Antibiotic Resistance Database (RGI v6.0.3, CARD v3.2.9.), utilizing the Strict and Perfect methods (Alcock et al., 2023). A heatmap of antimicrobial resistance (AMR) genes was created using the R pheatmap package (version 1.0.12) in RStudio. Capsule typing and outer core locus were identified via the Kaptive platform (Wyres et al., 2020; Cahill et al., 2022).

### Multilocus sequencing typing and eBURST analysis

The Oxford scheme was used for MLST analysis, querying the PubMLST database (https://pubmlst.org/organisms/acinetobacter-baumannii) (Bartual et al., 2005). The extraction of seven loci genes (*gltA*, *gyrB*, *gdhB*, *recA*, *cpn60*, *gpi*, and *rpoD*) from the 146 A*. baumannii* genomes was performed, indexed, and searched using makeblastdb and blastn with the BLAST suite software (version 2.9.0). Sequence types (STs) from previous studies were incorporated to illustrate ST diversity in Mexico. Clonal complexes (CCs) were analyzed with the Global Optimal eBURST (goeBURST) algorithm using PHYLOViZ 2.0 software (https://www.phyloviz.net/goeburst). The analysis of clonal complexes was conducted at the level of triple-locus variants (TLV) (Nascimento et al., 2017).

## Results

### Genetic diversity

In the analysis of diversity by ANI, eight distinct clusters were identified, each defined by specific ANI thresholds (**Supplementary Figure 1**). Two of these clusters contained pairs of genomes, while four clusters each comprised a single genome. Among the analyzed samples, two major genomic clusters were identified: the first cluster included 97 genomes, while the second contained 41 genomes. The high ANI values, exceeding 98%, underscored a significant degree of genomic consistency within these clusters.

The analysis revealed that these two major clusters were predominantly composed of genomes from specific geographic regions. The larger cluster included 75.2% (73/97) of samples collected from a single hospital in Jalisco between 2008 and 2018. The second cluster included 75.6% (31/41) of samples isolated from Mexico City (CdMex) between 2011 and 2018.

### Phylogenetic analysis

**Figure 1:**
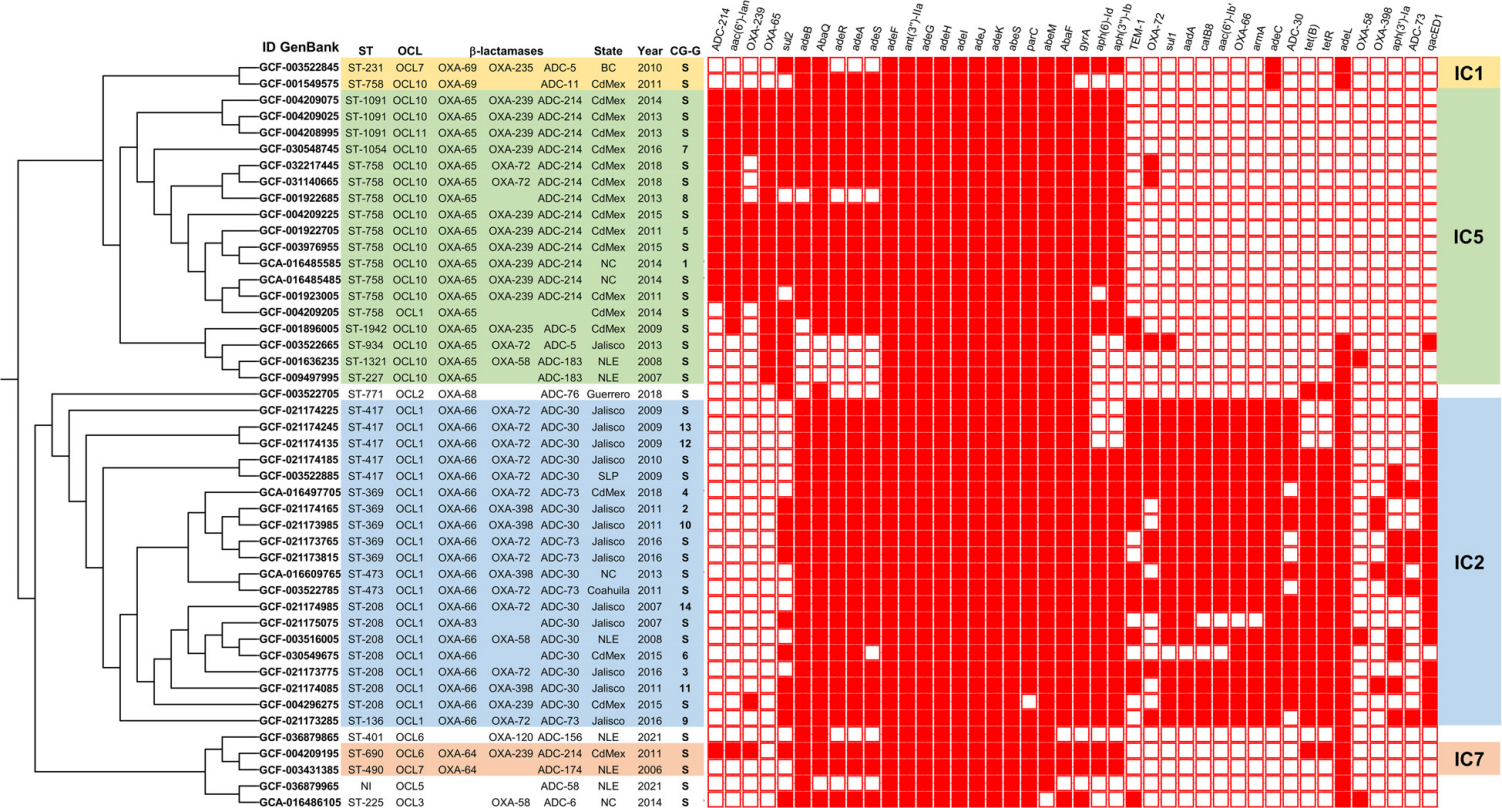
Phylogenetic tree displaying the relationships among 46 genomes within the clonal complexes (IC1, IC2, IC5, IC7). The tree is annotated with sequence types (STs), OCL-loci, β-lactamase genes, geographic locations, and years of isolation. The C/S column denotes the cluster (C) or (S) singleton. A heat map displays resistance gene profiles across the 46 genomes, where red indicates the presence of a resistance gene and white indicates its absence.

To prevent over-representation of closely related genomes between these two major groups observed in the ANI analyses, a core genome was made using all 146 A*. baumannii* genomes. This analysis identified 1,590 clusters of orthologous groups, with each cluster corresponding to a single gene across all genomes. Of these, 259 marker genes were used to generate a concatenated alignment. The algorithm identified clusters of similar core genomes among the 146 samples, which were represented as single branches in the phylogenetic tree (**Figure 1**). Therefore, the analysis categorized the genomes into 14 distinct clusters (C) each containing two or more genomes and identified 32 genomes as singletons (S). This resulted in a phylogenetic tree with 46 representative genomes out of the original 146 (**Supplementary Dataset 2**).

The isolates constituting each of the 14 clusters were obtained from specific locations; however, the number of genomes per cluster varied. The distribution was as follows: Cluster-1 with 2 genomes from an unspecified location; Clusters-2, 3, 8, 9, 10, 11, 12, 13, and 14 with genomes exclusively from Jalisco; and Clusters 4, 5, 6, and 7 with genomes from Mexico City (CdMex) (**Supplementary Dataset 2**).

**Figure 2:**
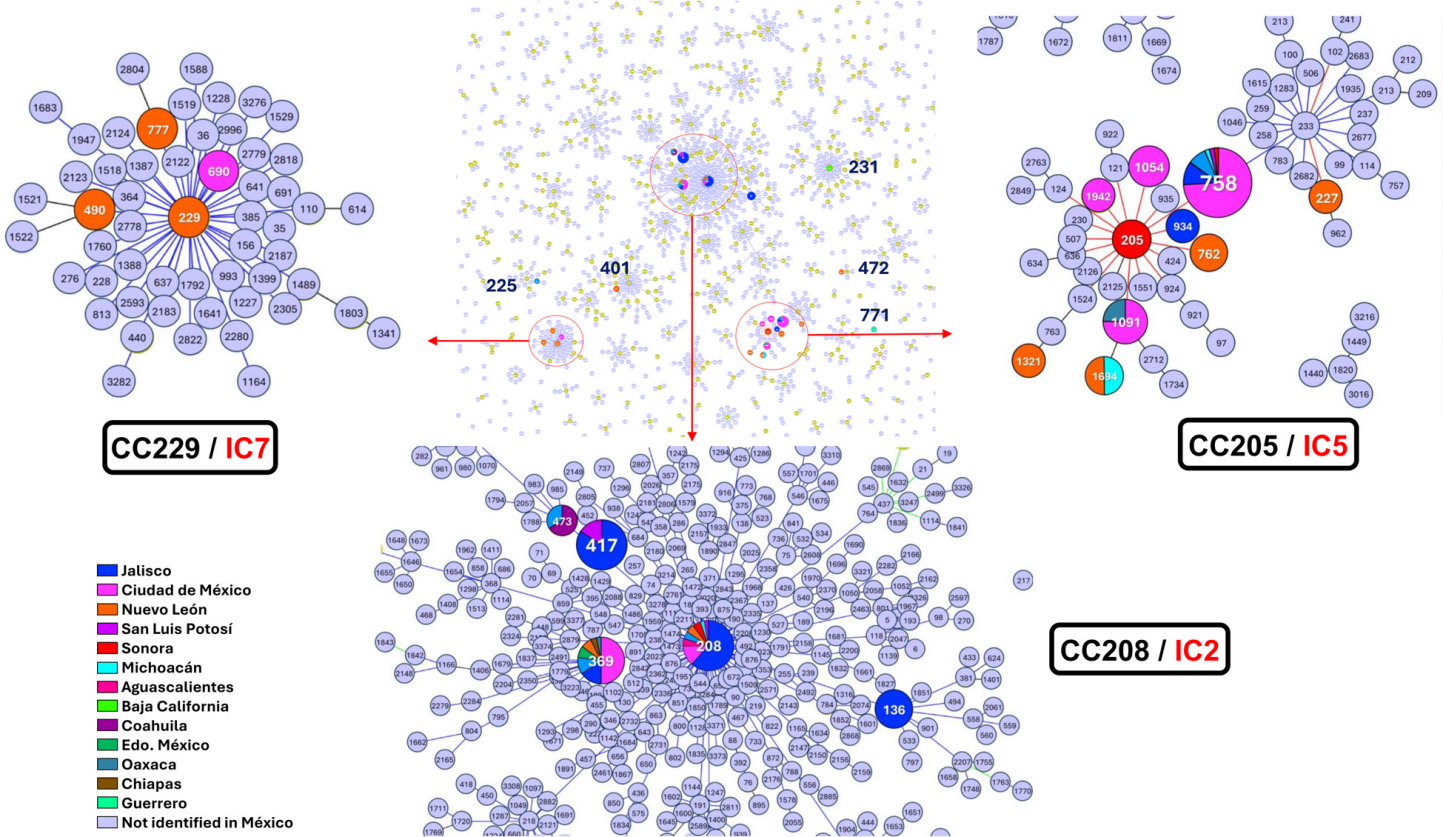
The Minimum spanning tree shows the relationships between different clonal complexes (CC) and their association with international clones (IC). Clonal complexes CC229 (IC7), CC205 (IC5), and CC208 (IC2) are highlighted, showing regional distribution across different Mexican states.

The analysis of the 46 genomes in the phylogenetic tree topology, identified two major phylogenetic groups, two groups with two genomes each, and four genomes without clear genetic relationships (GCF-036879965, GCF-036879865, GCF-003522705, GCA-016486105) (**Figure 2**).

The first major phylogenetic group consisted of 20 out of the 46 representative genomes. That group included Clusters 2, 3, 4, 6, 9, 10, 11, 12, 13, and 14 (totaling 86 genomes), as well as 10 singletons. The second major phylogenetic group consisted of 18 out of the 46 representative genomes. This group included Clusters 1, 5, 7, and 8 (totaling 28 genomes), along with 14 singletons. The two minor phylogenetic groups, consisting of two singletons each one and four genomes without clear genetic relationships (**Figure 1**).

To explore potential correlations between the phylogenetic tree topology and the genetic characteristics of the 146 genomes, ST assignment was performed using the Oxford MLST scheme. Additionally, capsule typing (KL), outer core locus (OCL) typing, and antibiotic resistance gene profiles were predicted.

Among the 146 genomes, we identified 18 distinct STs (ST136, ST208, ST225, ST227, ST231, ST369, ST401, ST417, ST473, ST490, ST690, ST758, ST771, ST934, ST1054, ST1091, ST1321, and ST1942). Capsule typing revealed 12 different KL types (KL1, KL2, KL7, KL9, KL22, KL23, KL32, KL48, KL77, KL169, KL195, and KL213), while 8 distinct OCL types were identified (OCL1, OCL2, OCL3, OCL5, OCL6, OCL7, OCL10, and OCL11) (**Supplementary Dataset 2**).

The phylogenetic tree also highlighted clear relationships among the groups, showing correlations between genetic traits and their distribution across the 14 clusters and the 32 singletons (**Figure 1**).

The first major phylogenetic group with 20 of the 46 representative genomes, predominantly comprised isolates from Jalisco 65% (13/20), where 95% (19/20) harbored β-lactamase OXA-66 and were predicted to possess the OCL1 locus. Additionally, the capsule typing revealed the presence of KL types KL2, KL9, KL22, KL23, KL77, and KL213. These genomes were assigned into sequence types ST136, ST208, ST369, ST417, and ST473 and were related to the major international clone IC2 according to Shelenkov et al., 2023.

The second major phylogenetic group consisted of 18 of the 46 representative genomes, predominantly included isolates from Mexico City 72.2 (13/18). The 100% (18/18) harbored β-lactamase OXA-65 and were predicted to possess the OCL10 locus. The capsule typing revealed the presence of KL types KL9, KL22, KL23, KL32, KL195, and KL213. These genomes were assigned to sequence types ST227, ST758, ST934, ST1054, ST1091, ST1321, and ST1942, and were closely related to the major international clone IC5.

One of the minor phylogenetic groups, consisting of two singletons from CdMex and Nuevo Leon (NLE), harbored β-lactamase OXA-64 and was predicted to have the OCL6 and OCL7 loci, with capsule typing revealing the presence of KL22 and KL7 types, respectively. These genomes were assigned to ST490 and ST690 and were related to the major international clone IC7. The other minor group, also consisting of two singletons from CdMex and Baja California (BC), harbored β-lactamase OXA-69 and was predicted to have the OCL7 and OCL10 loci, the KL1 was identified in both genomes. These genomes were assigned to ST231 and ST758 and were related to the major international clone IC1 (**Figure 2**). The genetic characteristics within these phylogenetic groups showed a high degree of conservation, including ST, OCL locus, KL locus, resistance gene profiles, and geographical location (**Supplementary Dataset 2**).

### Analysis of *in silico* prediction of ARGs

To investigate potential correlations between the major phylogenetic groups associated with international clones and their antibiotic resistance gene profiles, an *in silico* prediction of the resistome was conducted. This analysis was performed on the original 146 genomes and then plotted for the 46 representative genomes identified in the phylogenetic analyses.

The following genes were identified in all the genomes according to the CARD database: the *abeSM* (efflux pump system), *lpsB* (lipopolysaccharide), *AmvA* (efflux), *rsmA* (afflux pump), *ade* family (*adeCHLFGASBRJIKN*) (efflux complexes), *AbaQ* (efflux), *abeS* (efflux), *abaF* (efflux component), *ant(3’’)-IIa* (aminoglycoside), *gyrA* (quinolones) and *parC* (quinolones) (**Figure 1**).

Oxacillinase (OXA) genes belonging to the main family *bla*OXA-51-like were detected in 93.4% (43/46) of the genomes. However, the three genomes without OXA genes did not belong to the phylogenetic groups associated with the IC clones identified. According to the RGI and phylogenetic analysis, OXA-66 was found in 95% (19/20) of the genomes belonging to the phylogenetic group related to IC2 group, while OXA-65 was present in 100% (18/18) of the genomes belonging to the phylogenetic group related to IC5. Additionally, β-lactamases OXA-64 was present in the phylogenetic group related to IC7, and OXA-69 was found in the phylogenetic group related to IC1 (**Figure 1**).

In most of the genomes, a second oxacillinase was identified: the OXA-58, OXA-72, OXA-120, OXA-235, OXA-239, and OXA-398 were present in 6.5% (3/46), 32.6% (15/46), 2.7% (1/46), 4.3% (2/46), 26% (12/46), and 8.6% (4/46) of the genomes, respectively. Notably, 60% (12/20) of the genomes in the phylogenetic group related to IC2 had OXA-72, while 55.5% (10/18) of the genomes in the phylogenetic group related to IC5 had the OXA-239. Additionally, 19.5% (9/46) of the genomes did not harbor a second OXA gene. Most of the genomes 97.8% (45/46) were positive for the intrinsic *ampC* classified as a Class C β-lactamase. These enzymes are referred to as *Acinetobacter*-derived cephalosporinases (ADC). According to the phylogenetic tree, the two major phylogenetic groups were related to the main international clones IC2 and IC5. The presence of ADC-30 (15/20) and ADC-214 (13/18) was associated with the main international clones IC2 and IC5, respectively. Other ADC genes such as ADC-5, ADC-6, ADC-11, ADC-58, ADC-73, ADC-76, ADC-156, and ADC-174 were identified at lower frequencies, and in one genome, the ADC gene was not identified.

### Molecular epidemiology

To provide a comprehensive view of the molecular epidemiology of *A. baumannii* in Mexico, MLST analysis was performed to assign STs to 146 genomes, and STs reported in previous studies were integrated to illustrate the diversity of STs across the country. Major Clonal Complexes (CCs) were identified using the Global Optimal eBURST (goeBURST) algorithm, with analysis conducted at the level of Triple-locus variants (TLV) using PHYLOViZ 2.0 software. A total of 18 different STs were identified from the 146 genomes, and an additional six STs were obtained from previous reports.

The Minimum spanning tree (MST) analysis revealed three main CC. The CC205, include 10 different STs; from these the ST227, ST758, ST934, ST1054, ST1091, ST1321 and ST1942 were assigned from the genomes analyses, and the ST205, ST762 and ST1694 were obtained from previous reports. The ST205, ST758, ST762, ST934, ST1054, ST1091, and ST1942, were single-locus variants (SLVs) from each other. Additionally, this CC also contained sequence types ST227 which was an SLVs from the ST205. The ST1321, and ST1694 were double-locus variants (DLVs) distant from the other STs in this complex. The CC208 was composed of ST136, ST208, ST369, and ST473, which were SLVs of each other, the ST417 also was included as a DLV. The CC229 comprised ST229, ST490, ST690, and ST777, all of which were SLVs of each other. The ST490 and ST690 were assigned from the genomes analyses, and the ST229 and ST777 were obtained from previous reports.

Sequence types ST225, ST401, ST472, and ST771 were not associated with any of the main CCs identified in the analysis (**Figure 2**). However, current reports associate ST231 with the IC1 complex. The information about the STs was obtained from 13 different Mexican states, and the frequency of all STs is shown in (**Supplementary Figure 2**).

## Discussion

This study provides a comprehensive analysis of the molecular epidemiology of *A. baumannii* in Mexico using genomic data available up to July 2024. Our findings reveal significant genetic diversity among Mexican *A. baumannii* isolates and highlighting the prevalence of specific CCs related to four major ICs across different regions. According to the Oxford Scheme, 24 different sequence types were identified. Of these, 18 STs were assigned from the 146 genomes analyzed, while six additional STs were reported in previous studies (Bocanegra-Ibarias et al., 2015; Gonzalez-Villoria et al., 2016; Tamayo-Legorreta et al., 2014, 2016; Alcántar-Curiel et al., 2019; Garza-Ramos et al., 2023).

**Figure 3:**
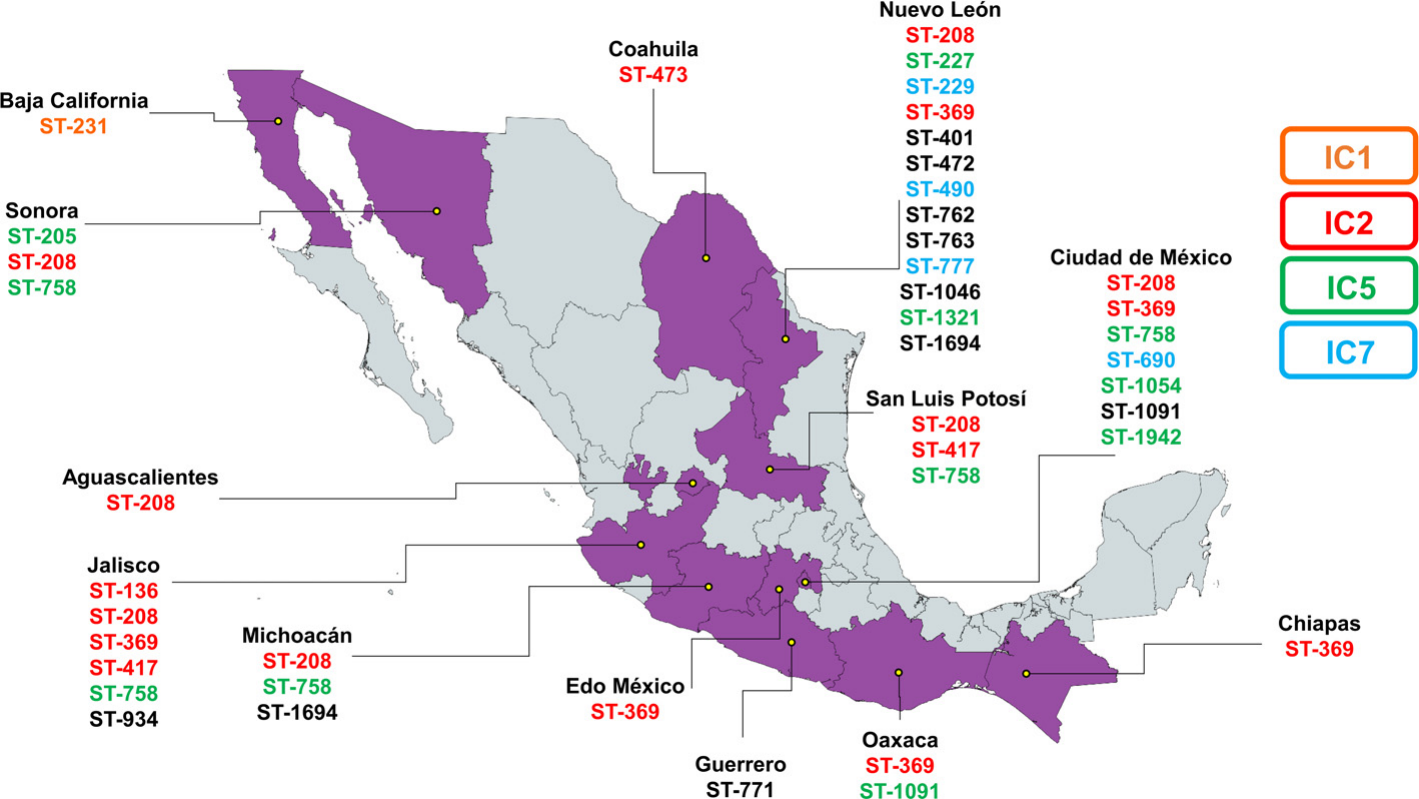
Geographic distribution of the most common sequence types across different Mexican states. Each ST is color-coded based on its association with an international clone.

In the analysis of the genetic characteristics of the main IC, it was observed that the phylogenetic group associated with the major international clone IC2 consisted of five different STs belonging to the CC208 (**Figure 2**). As previously reported, isolates belonging to IC2 harbor β-lactamase OXA-66, and in this case, almost all the genomes (95%) present this gene. Additionally, most of them also present β-lactamase ADC-30 at a high frequency (75%) and OXA-72 (60%) (Shelenkov et al., 2023). The prediction of OCL typing shows that all genomes belonging to CC208 and related to IC2 are predicted to possess the OCL1 locus. The OCL prediction can be used as a genetic marker for genome typing as previously suggested and useful to associated with specific IC (Wyres et al., 2020). It was observed that 65% of the samples were from the state of Jalisco; however, isolates from four others states also identified as part of IC2. This suggests that the isolates conforming to IC2 were not exclusive to a specific period or region of Mexico and are likely to be widely distributed in other regions of the country (**Figure 3**).

The second major phylogenetic group identified present highly diversity, consisting of 10 STs assigned to the CC205, which is related to the major international clone IC5. All the genomes within IC5 harbor β-lactamase gene OXA-65, with β-lactamase ADC-214 present in 72.2% of the genomes and OXA-239 in 50%. OCL prediction showed that most of the genomes (82.2%) possess the OCL10, while OCL1 and OCL11 were predicted in two genomes. The 72% of the samples were from CdMex but isolates from two other states were also identified as part of IC5, indicating that IC5 is not confined to a specific region in Mexico.

One of the minor phylogenetic groups consisted of two singletons but included four STs (two ST from previous reported) assigned to the CC229 and were related to the international clone IC7. Most of the samples originated from Nuevo Leon (NLE) and one from CdMex. The β-lactamase OXA-64 was identified in both singletons, with ADC174 and ADC214 found in each genome, and OCL prediction indicated OCL6 and OCL7 loci. To fully establish the genetic characteristics of IC7, it will be necessary to use a high number of genomes in the analysis, However, the presence of OXA-64 and assignment to CC229 provide reasonable evidence of a correlation with IC7.

The other minor group, consisting of two singletons from Baja California (BC) and CdMex, isolated in 2010 and 2011, respectively. Despite belonging to different STs (ST231 and ST758), these genomes were clustered in the same clade after phylogenetic analysis. Both genomes harbored β-lactamase OXA-69; ADC5 was identified in ST231 and ADC11 in ST758. The OCL prediction revealed that OCL7 and OCL10 loci were present in ST231 and ST758, respectively. Although only one genome was assigned to CC231, and despite the differences in ADC gene and OCL loci, the presence of β-lactamase OXA-69 in both genomes and their clustering in the same group after phylogenetic analysis strongly suggests that both genomes share the same genetic background and likely associated with international clone IC1 (**Figure 2**).

However, the correlation between the ST assignment using the Oxford MLST scheme and phylogenetic analysis does not always align perfectly in the molecular epidemiology of *A. baumannii*. For instance, in the analysis, one of the two genomes grouped into CC231 was assigned as ST758 by the Oxford MLST scheme. Both genomes shared the β-lactamase OXA-69 gene, clustering within CC231, and correlate with the IC1 after phylogenetic analysis. Despite such discrepancies, the Oxford MLST scheme remains a reliable tool for molecular epidemiology. In fact, the results of this study demonstrated strong concordance between the STs assigned to specific CCs and their phylogenetic groups based on core genome analysis, which also correlated with major international clones.

There is ongoing debate about which MLST scheme is the most appropriate for studying the molecular epidemiology of *A. baumannii*, evidence suggests that the Oxford MLST scheme has greater discriminatory power than the Pasteur scheme and is more effective at identifying the main international clones (Tomaschek et al., 2016; Castillo-Ramírez and Graña-Miraglia, 2019; Hua et al., 2020). This observation was further confirmed by Gaiarsa et al., who found that the Oxford MLST scheme has greater discriminatory ability and concordance than the Pasteur MLST scheme, even though the authors suggested that the Pasteur scheme might be preferable for certain epidemiological studies of *A. baumannii* (Gaiarsa et al., 2019).

This controversy has been addressed by suggesting that data obtained from modern technologies, such as whole-genome sequencing (WGS) and the core-genome analysis, provides a better comprehensive understanding of the current epidemiological landscape (Moreno-Manjón et al., 2023; Müller et al., 2023). There is no doubt that WGS has become a fundamental tool in molecular epidemiology, significantly enhancing the understanding of clone spread, virulence, and antibiotic resistance in pathogenic bacteria. However, the Oxford MLST scheme remains valuable, offering a standardized and accessible method for tracking the global distribution and epidemiology of *A. baumannii*. Rather than being excluded, the Oxford MLST scheme continues to play an important role alongside newer technologies, particularly in settings, where resources for WGS may be limited or in studies where comparability to previous data is crucial.

In conclusion, the identification of the main international clones IC1, IC2, IC5 and IC7 were obtained by the phylogenetic analysis, their genetic characteristics correlated with high accuracy with the assigned ST and CC. Additionally, the use of outer core locus (OCL) and capsule locus (KL) typing, as previously suggested offers valuable tools for studying the clinical epidemiology of *A. baumannii* (Wyres et al., 2020). These methods show potential as complementary genetic markers for correlating with major ICs. However, further extensive analysis will be needed before they can be established as a standard genetic marker. Nevertheless, OCL prediction remains promising as a useful supplementary tool for IC designation.

In recent years, genomic epidemiology studies in Mexico have primarily focused on clone dissemination within specific hospitals and cities. However, to fully understand the dynamics and high diversity of *A. baumannii* clones to spreading within hospitals and across regions, a more comprehensive epidemiological analysis is necessary. This should include isolates from most Mexican states, covering different years, to better understand the persistence and dissemination patterns of the main clonal complexes and international clones.

Understanding the epidemiology of *A. baumannii* at a national level is essential for constructing a comprehensive global perspective. The identification of four major international clones in Mexico emphasizes the importance of conducting localized studies to elucidate the complex dynamics and variations in the dissemination of *A. baumannii* patterns. Global studies like Müller et al. provide valuable insights into the distribution of international clones and carbapenemase genes, they rely on detailed, country-specific data to fully understand local epidemiological patterns (Müller et al., 203). These localized studies are essential for understanding the complexities of resistance mechanisms and clone prevalence, allowing for improved public health interventions and effective outbreak management.

## Data Availability

The datasets presented in this study can be found in online repositories. The names of the repository/repositories and accession number(s) can be found in the article/**Supplementary Material**.

## Supplementary material

Supplementary Figure 1 Heatmap of Average Nucleotide Identity (ANI) from 146 A*.baumannii* genomes, clustered by international clones (IC1, IC2, IC5, and IC7). The intensity of the color represents the degree of nucleotide identity, with red indicating high similarity. The clustering dendrograms on the axes show the phylogenetic relationships between the isolates.
